# Effect of high-quality pellet feed level on voluntary feed intake, nutrient digestibility and rumen fermentation in beef cattle

**DOI:** 10.1038/s41598-025-96455-z

**Published:** 2025-05-02

**Authors:** Thitima Norrapoke, Tanitpan Pongjongmit

**Affiliations:** Department of Animal Production Technology, Faculty of Agricultural Technology, Kalasin University, Meang Kalasin, 46000 Kalasin Province Thailand

**Keywords:** Cassava pulp, Pellet feed, Rumen fermentation, Methane production, Ecology, Ecosystem ecology

## Abstract

The aim of this study was to evaluate the effect of high-quality pellet feed on voluntary feed intake, nutrient digestibility and rumen fermentation in beef cattle. Four beef cattle aged approximately 2–3 years were randomly assigned according to a 4 × 4 Latin square design to compare the replacement of pelleted diets in concentrated diets at 4 levels: 0% of diets (T1), 20% of diets (T2), 40% of diets (T3) and 60% of diets (T4). The results of this study revealed that body weight change rate/day total edible amount, or the amount of feed that can be eaten out of concentrated feed, was not significantly different (*P* > 0.05) among the beef cattle fed all 4 treatments. The digestibility of dry matter, organic matter, crude protein, NDF and ADF was highest in pelleted-fed beef cattle, at 60%. However, the combination of a pelleted diet and a concentrated diet resulted in a statistically significant reduction in protozoa populations when the pelleted diet level was increased. The level of pelleted feed had no effect on the pH or rumen temperature of the beef cattle. With respect to ammonia nitrogen, the propionic acid, butyric acid and total volatile fatty acid levels increased when the beef cattle were fed more pelleted feed. especially at 60%, but the acetic acid and methane production decreased when the beef cattle were fed higher levels of pelleted feed. Purine derivative release was not significantly different. In addition to purine derivative absorption and microbial nitrogen supply, the efficiency of microbial protein synthesis was greater when beef cattle were fed high-quality pelleted feed than when they were not fed high-quality pelleted feed. The present study concluded that pellet feeding in conjunction with concentrated feed at 60% resulted in decreased methane production, protozoa population and nutrient digestibility, microbial protein synthesis, total volatile fatty acids and propionic acid.

## Background

The level of high-quality pellet feed in the diet of beef cattle plays a critical role in determining voluntary feed intake, nutrient digestibility, and rumen fermentation processes. High-quality pellets, typically formulated with a balanced mix of essential nutrients, are designed to meet the dietary requirements of cattle, promoting growth and productivity. However, the appropriate level of pellet inclusion is crucial to optimize feed efficiency and overall animal health^1^. Understanding how varying levels of these pellets influence key aspects of digestion and metabolism is essential for developing feeding strategies that enhance cattle performance.

Voluntary feed intake is a primary factor influencing the nutritional status and energy balance of cattle. The inclusion of high-quality pellets can impact how much cattle consume, as well as how effectively they digest and absorb nutrients from their diet^2^. Additionally, the process of rumen fermentation, where microbes break down feed to produce volatile fatty acids and other essential nutrients, is significantly affected by diet composition. The balance between different types of feeds, including high-quality pellets, can alter rumen pH, fermentation end-products, and microbial populations, ultimately influencing cattle productivity^3^.

This study aims to investigate the effect of different levels of high-quality pellet feed on voluntary feed intake, nutrient digestibility, and rumen fermentation in beef cattle. By exploring these relationships, we can identify optimal feeding practices that maximize nutrient utilization and improve the efficiency of beef production. The findings will contribute to a better understanding of how dietary adjustments can enhance the health and performance of beef cattle, with implications for both animal welfare and agricultural sustainability.

By encouraging the development of ruminal microorganisms, we hypothesized that high-quality pellets feed could be useful in modulating rumen fermentation. Therefore, this research aims to study the effects of high-quality pellets feed on feed intake, rumen fermentation and microbial population in beef cattle. Therefore, nutrient digestibility and fermentation in the rumen of beef cattle is an interesting approach. Locally available raw materials for animal feed should be utilized, as well as increased feed management efficiency.

## Materials and methods

All the research was carried out in accordance with the principles and regulations approved by Kalasin University’s animal husbandry and use ethical committee. Sources of cassava pulp were obtained from Kalasin Province.

### Feed and preparation of experimental feeds

Preparing compressed feed by using fermentation residues (following the method of^4^ before using them to make compressed feed), cassava chips, rice bran, premix, molasses, and salt, compressing them into the size of compressed feeds available in the industry. The cassava pulp was purchased.

### Experimental design, animals, and treatment

In this experiment, four brahman crossbreed beef cattle with beginning body weights (BWs) of 300 ± 40 kg were used. The beef cattle used in this study were obtained from Kalasin University Framework. Each animal was housed in its own pen and was fed a concentrated diet (14% CP). Diets consisted in a concentrate, offered at 1.5% of BW registered at start of each period. Rice straw (no treatment), water, and mineral salt blocks were provided ad libitum. The experiment used a 4 × 4 Latin square design to compare the replacement of pelleted diets in concentrated diets at 4 levels: 0% of diets (T1), 20% of diets (T2), 40% of diets (T3) and 60% of diets (T4). The chemical composition is shown in Table 1. The experiment was carried out during four distinct periods, each of which lasted 21 days. All animals were fed their respective diets for the first 14 days as a time of adaptation, after which a 7-day collection period was used.

### Data collection and sample analysis

For body weight gain measurements, animals were measured at the start and finish of each period. Throughout the experimental periods, feed intake was monitored and calculated by deducting the amount of feed refused from the amount of feed offered to the animals, with the leftover feed discarded before the morning feeding of high-quality pellet feed. Concentrations and rice straw samples were adjusted throughout the final seven days of each period. Rectal sampling was used to collect the feces, and a spot sampling technique was used to collect the urine. The obtained samples were dried at 72 °C, milled (1 mm screen using a Cyclotech Mill, Tecator, Sweden), and measured for dry matter (DM), ash, ether extract (EE), and crude protein (CP) by the protocols of the Association of Official Analytical Chemists^5^. According to Van Soest et al.^6^, analyses of NDF and ADF were carried out using amylase without sodium sulfite, acid insoluble ash (AIA) or residual ash and were included in the expression of NDF and ADF^7^.

Digestion can be calculated by determining the difference between the nutrients consumed and the nutrients excreted by the animal. This process involves measuring the total feed intake and analyzing its nutrient composition, such as dry matter, organic matter, crude protein, and fiber components like neutral detergent fiber (NDF) and acid detergent fiber (ADF).

The feces produced by the animal are collected and analyzed for the same nutrients to determine how much of the feed was not digested and excreted. The digestibility is then calculated as the proportion of nutrients that were absorbed and utilized by the animal.

The formula for digestibility is expressed as a percentage:

$${\text{Digestibility}} (\%)=({\text{Nutrient}} \, {\text{Intake}} -{\text{Nutrient }}\, {\text{Excreted}} /{\text{Nutrient }}\, {\text{Intake}}) \times 100$$

For more specific calculations, the digestibility of organic matter can be used to estimate digestible organic matter intake (DOMI) and digestible organic matter fermented in the rumen (DOMR). These values help in understanding how effectively the feed supports microbial fermentation and the energy available to the animal.

Blood samples (approximately 10 ml) were drawn from the jugular vein on the last day of the data collection period at 0, 3 and 6 h after feeding and placed in tubes containing 12 mg of EDTA. The plasma was then spun at 5000 × g for 10 min to separate it (Table Top Centrifuge PLC-02, USA). The collected plasma was kept at 20 °C until blood urea nitrogen (BUN) and hemoglobin content (Hct) were measured in accordance with methods described in^8,9^, respectively. On the final day of the data collection session, rumen fluid samples were taken at 0, 3 and 6 h after feeding. The oral stomach collecting system included a 15 ml perforated plastic conical tube that was attached to one end of 90 cm polyvinyl chloride orogastric tubing and served as the rumen sieve. The other end of the stomach tube was linked to an electrical vacuum pump. Each time, a stomach tube attached to a vacuum pump was used to extract approximately 50 ml of rumen fluid from the rumen center. The pH and temperature of the rumen fluid were quickly determined using a portable pH and temperature meter (HANNA HI-8424 Portable pH/ORP Meter, Woonsocket, USA). After that, the rumen fluid samples were filtered through four layers of cheesecloth. To stop the microbial fermentation process, 45 ml of rumen fluid was collected and kept in a plastic bottle with 5 ml of sulfuric acid solution (1 M), and the mixture was centrifuged for 15 min at 16,000 g (Table Top Centrifuge PLC-02, FL, USA). High-performance liquid chromatography (HPLC) was used to separate the volatile fatty acids (VFAs), as described previously^10^. The ammonia nitrogen concentration in the supernatant (NH_3_-N) was measured. One milliliter of ruminal fluid was combined with nine milliliters of formalin solution as the final stage. Bacteria, protozoa, and fungi were subsequently counted under a microscope and hemocytometer (Boeco, Hamburg, Germany).

#### Calculations

Digestible organic matter fermented in the rumen (DOMR) was calculated as follows: DOMR (kg/d) = digestible organic matter intake (DOMI, kg/d) × 0.65, where DOMI = [digestibility of organic matter (kg/kg DM) × organic matter intake (kg/d)]/100, 1 kg DOMI = 15.9 MJ ME/kg^11,12^.

For determining microbial population, protozoa and fungus were enumerated under a microscope and a hemocytometer^13^. Quantities was expressed as cell/mL.

Microbial purines concentration and the efficiency of microbial N synthesis were measured using purine derivative excretions according to equation developed by Chen and Gomez^14^.

$${\text{Y}}=0.12{\text{X}}+(0.20{\text{BW}}0.75)$$

Microbial N synthesis was estimated by urinary estimated by urinary excretion of purine derivatives (PD) according to the equation of^14^:

$${\text{MN (g/d)}}=70 {\text{X}} /(0.116 \times 0.83\times 1000)=0.727{\text{X}}$$

Where X and Y are, respectively, absorption and excretion of PD in mmol/d. Efficiency of microbial N synthesis (EMNS) was calculated using the following formula:

$${\text{EMNS}}={\text{microbial}} \, {\text{N (g/d)}} / {\text{DOMR}}$$.

Where DOMR = digestible OM apparently fermented in the rumen.

#### Quantification of the microbial population

To recover community DNA from 0.5 g of rumen fluid and digesta, Yu and Morrison’s^15^ repeated bead beating plus column (RBB + C) method was used, and this method was found to significantly increase DNA yields. A total of sixteen samples were taken at two different intervals to sample rumen fluid (0 and 6 h postfeeding) from four treatments and four time periods. The quantity and quality of each of these DNA samples were evaluated using spectrophotometry and agarose gel electrophoresis^15^. To construct a quantitative test, the target 16 S rDNA of each species was amplified using specific primers, and the purified DNA was quantified using a spectrophotometer and several dilutions. Sequential tenfold dilutions of the previously measured DNA standards, ranging from 10^3^ to 10^9^ DNA copies, were used to measure the target DNA. Real-time PCR was performed for amplification and detection using a Chromo 4™ instrument (Bio-Rad, USA).

The standard PCR procedure for *F. succinogenes* was as follows: In the first cycle, there was 9 min of denaturation, and in the last cycle, there was 10 min of extension. All the other details of the amplification of the other two 16 S rRNA genes were similar, except for the annealing temperature, which was set at 55 °C. The quantification of primer numbers, status, and total bacterial counts was performed as previously described^15^. Four standards obtained from the samples were developed with the community’s DNA processing pool set. DNA standards were generated from samples using standard PCR procedures for each real-time PCR test. A spectrophotometer was subsequently used to measure the PCR products, and a QIA Rapid PCR Purification Kit (QIAGEN, Inc., Valencia, CA) was used to purify the products. The copy number concentration for each standard generated from the sample was determined using the mass concentration and length of the PCR product. Prior to real-time PCR, tenfold serial dilutions were performed in Tri-EDTA^15^. In total, four real-time PCR standards were created. The target gene real-time PCR experiment used the same settings as those previously described for conventional PCR. The Biotools QuantiMix EASY SYG Kit (B&M Labs, S.A., Spain) was used for real-time PCR amplification. PCR was subsequently conducted twice.

### Statistical analyses

Using the Proc GLM/Proc Mix, the data were statistically analyzed using a 4 × 4 Latin square design by using the SAS^16^. The data were analyzed using the model Yijk = µ + Mi + Aj + Pk + εijk, where Yijk is the observation from animal j receiving diet i in period k; µ is the overall mean; Mi is the effect of treatment (i = 1 to 4); Aj is the effect of the animal (j = 1 to 4); Pk is the effect of period (k = 1 to 4); and εijk is the residual effect *p* < 0.05 indicated statistical significance. All the data were subjected to variance analysis. Using orthogonal polynomial contrasts, the treatment trends were statistically contrasted (linear and quadratic). Tukey’s test was performed to determine differences between treatment means, and *p* < 0.05 was regarded as statistically significant.

#### Results and discussions

The chemical compositions of the concentrate, pellet feed and rice straw used in the experiments are shown in Table 1. The chemical composition of the concentrate, pellet feed and rice straw contained CP at 26.08, 14.03 and 2.77% DM; NDF at 38.12, 45.52 and 70.32% DM; and ADF at 25.15, 21.62 and 47.22% DM, respectively.

**Table 1:** Chemical composition of the concentrate, pellet feeds and rice straw.

Items	Pellet feeds	Concentrate	Rice straw
Cassava chip	20		
Cassava pulp	47		
Rice bran	20		
Urea	5		
Molasses	5		
Sulfur	1		
Premix	1		
Salts	1		
Chemical composition			
DM, %	94.53	95.23	96.71
	% dry matter		
Ash	8.12	11.12	10.11
OM	91.88	88.88	89.89
CP	26.08	14.03	2.77
AIA	1.23	1.55	5.45
NDF	38.12	45.52	70.32
ADF	25.15	21.62	47.22

*RS * rice straw, *DM * dry matter, *OM * organic matter, *CP * crude protein, *AIA * acid insoluble ash, *NDF * neutral detergent fiber, *ADF * acid detergent fiber.

The provision of supplemental concentrated feed influences production efficiency. Table 2 presents the quantities consumed. The daily rates of change in body weight, total feed intake, roughage intake, and concentrate intake were not significantly different (*P* > 0.05) among the cattle from butchers that were fed the four experimental diets. This is similar to what Pongjongmit et al.^17^ found when they looked at the nutritional value of a cassava flour fermentation supplement and cassava residue from a noodle factory. Beef cattle consumed a certain quantity of feed. Beef cattle receiving a cassava flour fermentation supplement combined with noodle mill residue exhibited no significant variation in their feed intake.

Supplementary feeding in conjunction with concentrated feed is a prevalent method to improve production efficiency in beef cattle. The study’s results show that there were no significant differences (*P* > 0.05) in the performance measures of the cattle that were fed the four different diets. These measures included daily body weight change, total feed consumption, roughage intake, and concentrate intake. This indicates that the supplementing technique utilized did not significantly affect the growth performance or feed intake behaviors of the cattle in this investigation.

Various factors may account for the absence of notable differences in these parameters. A potential explanation is that the nutritional composition of the supplementary and concentrated feeds was adequate to fulfill the animals’ dietary needs, resulting in a plateau effect where further supplementation did not improve performance^18^. The palatability and digestibility of the feeds utilized may have been comparable across the various diets, leading to uniform feed intake and growth rates in the cattle^19^. These results agree with those from other studies that show that extra feeding can make cattle more productive under certain circumstances. However, the exact nutrients, the ratio of roughage to concentrate, and the overall diet formulation^20^ all have a big effect on how well it works. Consequently, although supplementary feeding is a significant asset in beef cattle production, it is imperative to customize the feeding strategy according to the particular requirements of the cattle and the accessible feed supplies to attain the best outcomes.

**Table 2:** Effects of high-quality pellet feed on beef cattle production efficiency and feed intake.

Items	Level of pellet feed in diet (%)				SEM	Contrast	
	0	20	40	60		Linear	Quadratic
BWG, kg/d	1.46	1.35	1.40	1.44	0.41	0.99	0.85
Total DM intake							
kg/day	8.13	7.98	8.07	8.21	1.13	0.95	0.90
%BW	2.65	2.62	2.60	2.55	0.25	0.78	0.96
g kg^− 1^BW^0.75^	110.68	109.36	108.78	107.77	11.40	0.86	0.98
Rice straw intake							
kg/day	3.72	3.61	3.54	3.69	0.30	0.90	0.68
%BW	1.22	1.19	1.16	1.16	0.08	0.59	0.89
g kg^− 1^BW^0.75^	50.76	49.61	48.35	48.93	3.15	0.65	0.79
Concentrate intake							
kg/day	4.41	4.38	4.54	4.52	0.85	0.90	0.99
%BW	1.43	1.43	1.44	1.39	0.19	0.88	0.91
g kg^− 1^BW^0.75^	59.93	59.76	60.44	58.84	8.94	0.95	0.94

*BWG * Body weight gain, *SEM* Standard error of the means.

^ab^Values in the same row with different superscripts differ (*P* < 0.05).

Table 3 illustrates the impact of dietary supplements in conjunction with concentrated feed on the digestibility of beef cattle. The investigation showed that pellet-fed beef cattle maximized the digestibility of organic matter and NDF at 60%; however, the digestibility of dry matter, crude protein, and ADF did not show significant differences among treatments. The presence of fiber from cassava pulp, particularly NDF, may contribute to the excellent quality of pellet feed. Pongjongmit et al.^17^ found that the nutritional value of voluntary feed intake and digestibility in beef cattle were affected by fermentation supplements made from cassava pulp and noodle leftovers. Beef cattle were identified as an alternative source for fermenting cassava pulp and noodles industry byproducts. We observed no significant change in digestibility.

The findings, illustrated in Table 3, indicate that nutritional supplementation alongside concentrated feed has a varying effect on the digestibility of several components in beef cattle. Cattle consuming a diet of 60% high-quality pellet feed maximized the digestibility of organic matter (OM) and neutral detergent fiber (NDF). We identified no significant variations in the digestibility of dry matter (DM), crude protein (CP), and acid detergent fiber (ADF) across the treatment groups. The improved digestibility of OM and NDF in calves consuming the 60% pellet feed diet is due to the pellets’ composition, notably the incorporation of cassava pulp. Cassava pulp is recognized for its elevated NDF concentration, which likely enhanced the fiber digestibility noted in this treatment group^21^. NDF quantifies the components of plant cell walls, such as cellulose, hemicellulose, and lignin, which are essential for rumen fermentation and overall digestive efficacy. Adding cassava pulp to the premium pellet feed probably made it a more fermentable fiber source, which led to more microbes in the rumen and better digestion of these nutrients^22^.

Regardless of the dietary supplementation dose, the digestibility of dry matter, crude protein, and acid detergent fiber remained relatively unchanged across treatments. This may result from the sufficient protein and energy levels supplied by the concentrated feed, which fulfilled the cattle’s nutritional needs irrespective of the differing degrees of pellet supplementation^23^. These findings underscore the necessity of evaluating the precise composition of dietary supplements in the formulation of cattle diets. The incorporation of high-quality pellet feeds, particularly those with cassava pulp, can improve fiber digestibility; however, the effect on other nutritional elements may be negligible if the foundational diet is already well-balanced. Therefore, the strategic application of these supplements should prioritize the enhancement of fiber utilization while maintaining the digestibility of other vital nutrients.

Accurate digestion data significantly enhances the clarity and impact of result descriptions in research. When the data is correct, it provides a reliable foundation for understanding how nutrients are utilized by animals, allowing for precise comparisons across dietary treatments. This accuracy enables researchers to highlight differences in nutrient digestibility and link them to animal performance metrics, such as growth or productivity. Moreover, it supports a clearer explanation of the physiological and environmental impacts, such as the reduction of methane emissions with improved feed efficiency. Reliable digestion data also strengthens statistical analyses, making the findings more credible and scientifically robust. Ultimately, accurate data allows for practical, evidence-based recommendations that are actionable for farmers and beneficial for livestock management.

**Table 3:** Effects of high-quality pellet diets on the digestibility of beef cattle.

Items	Level of pellet feed in diet (%)				SEM	Contrast	
	0	20	40	60		Linear	Quadratic
Apparent digestibility, %							
Dry matter	48.13	45.75	56.86	55.32	8.81	0.43	0.96
Organic matter	30.13^b^	34.11^ab^	35.20^ab^	39.22^a^	2.22	0.02	0.99
Crude protein	30.55	31.55	30.13	32.17	4.83	0.88	0.92
Neutral detergent fiber	28.35^b^	32.78^ab^	33.58^ab^	44.98^a^	3.93	0.02	0.39
Acid detergent fiber	24.30	29.07	26.49	30.36	2.92	0.26	0.88

^ab^ Values in the same row with different superscripts differ (*P* < 0.05).

*SEM* Standard error of the means.

We supplemented the diet with feeds that targeted blood urea nitrogen (BUN) and hematocrit levels. Table 4 displays the hematocrit levels in beef cattle prior to eating and after a 4-hour feeding period. The trial demonstrated that, among beef cattle subjected to all four treatments, the mean BUN values exhibited no significant differences. The protein level of beef cattle, when combined with concentrated feed, may not meet their requirements, resulting in a diminished absorption of NH3-N through the rumen. This does not indicate that the BUN concentration is excessively high or low, since it falls between 8.55 and 9.46 mg/dL, which is within the usual range of 7 to 20 mg/dL^24^. BUN levels vary according to several parameters, including age, protein consumption, and particularly ammonia nitrogen concentration in the rumen. An elevation in ammonia nitrogen levels in the rumen leads to an increase in blood urea nitrogen (BUN) levels^25^. The BUN concentration functions as a gauge of nitrogen consumption, while the levels of edible nitrogen and hematocrit do not significantly alter, staying within the typical range of 25–29%. This finding indicates that the health of the beef cattle is normal and devoid of anemia. Norrapoke et al.^26^ similarly investigated the application of urea in conjunction with fermented molasses. Cassava determined that the hematocrit levels ranged from 28.42 to 29.17%. The hematocrit serves as an index of red blood cell count or platelet density^27^.

The study looked at how different diets affected blood urea nitrogen (BUN) and hematocrit levels in beef cattle. The average BUN values did not change significantly between the four treatment groups. The BUN readings varied from 8.55 to 9.46 mg/dL, falling within the usual range of 7 to 20 mg/dL for healthy cattle^28^. In other words, the concentrated feed’s protein content wasn’t higher than what the cattle needed, which kept the rumen from absorbing too much ammonia nitrogen (NH3-N).

The cattle’s adequate protein intake, which met but did not exceed their metabolic requirements, is responsible for the consistent BUN levels observed across treatments. The fact that BUN levels are linked to ruminal ammonia production and protein metabolism suggests that the animals are using nitrogen effectively, which means that their metabolic processes are not being overworked^23^. The absence of significant differences in BUN levels indicates that the diets did not result in excessive ammonia accumulation in the urine, which could otherwise increase BUN concentrations and potentially cause metabolic disturbances. In addition to BUN, the study measured hematocrit levels before and four hours after feeding. Hematocrit levels remained steady and within the normal range of 25–29% in all treatment groups, showing that dietary interventions had no substantial impact on red blood cell concentration. Normal hematocrit levels signify the cattle’s proper hydration and absence of anemia or other blood-related health issues^29^. These data combined indicate that the nutritional supplementation options used in this investigation were effective in sustaining normal physiological health in beef cattle. The consistent BUN and hematocrit values across all treatment groups support the notion that the feeds offered enough nutrition while minimizing metabolic stress. As a result, these values are important markers for monitoring the protein and nitrogen balance in cattle, ensuring that dietary adjustments promote optimal health and productivity while minimizing negative impacts.

Table 4 illustrates the impact of supplementary feed and concentrated feed on the ruminal fermentation of beef cattle. Experiments indicated that the concentration of feed pellets did not influence the pH or temperature in the rumen of beef cattle. The levels of ammonia nitrogen, propionic acid, butyric acid, and volatile fatty acids increased with higher pellet feeding in beef cattle, particularly at 60%. Conversely, acetic acid and methane production decreased with increased pellet feeding. The replacement of concentrate with pellets may result in unequal nutrient distribution, particularly in terms of crude protein, for the cattle. A previous study reported that cassava pulp and cassava chips contain condensed tannins that may affect methane production. In alignment with the findings of Cherdthong et al.^30^, Thai beef cattle fed chopped fresh cassava roots at 1.5% of their body weight exhibited higher C3 values compared to those receiving cassava roots at 1% of their body weight. It is feasible to extract increased amounts of starch from cassava tubers, which serve as a source of nonstructural carbohydrates that act as precursors for C3 production. This finding fits with the work of Sommai et al.^31^, who found that eating more fermented cassava flour led to higher levels of TVFA and C3, but a linear drop in C2:C3 and CH4 values. Moss^32^ indicated that dietary modifications involving sodium hydroxide or ammonia, along with the addition of protein to low-quality feeds, led to a reduction in methane production per kilogram of edible feed. Additionally, Shioya et al.^33^ presented findings from their investigation of a feeding strategy. Ruminant agriculture in South Asia. Utilize sweet potatoes in conjunction with low-quality hay. We observed a decrease in methane production from 260 to 146 L per day and from 48.1 to 25.5 L per kilogram of standard milk (4% fat-corrected milk, FCM) in dairy cows fed exclusively hay and a combination of hay and sweet potatoes.

The study looked into what happened to beef cattle’s ruminal fermentation when they were fed both concentrated feed and extra feed. The results are shown in Table 4. The results show that changing the amount of feed pellets didn’t have a big effect on the rumen’s pH or temperature. This means that adding pellets kept the rumen’s environment stable at all treatment levels. The maintenance of stable pH and temperature is essential for maximizing microbial activity and enhancing digestive efficiency in ruminants^34^. The study discovered that the amounts of ammonia nitrogen (NH3-N), propionic acid, butyric acid, and total volatile fatty acids (VFAs) went up when the pellets were fed at higher levels, especially when 60% of the pellets were included. It’s likely that the feed pellets provided a readily fermentable substrate that helped the production of these important fermentation end-products, which is why the amounts that were seen went up. Propionic acid is a crucial precursor for glucose synthesis in ruminants, playing a significant role in energy metabolism and potentially enhancing growth performance^35^. The study indicated that an increase in pellet concentration resulted in a decrease in the production of acetic acid and methane. Acetic acid is often connected to the fermentation of fibrous feeds. A drop in its concentration could mean that the fermentation profile is changing to one that is dominated by starch, which is usually accompanied by increased pellet feeding^36^. This shift may significantly impact methane production, given the correlation between acetate production and increased methane emissions. Increasing pellet feeding leads to less methane production and less acetic acid production. This suggests that reducing enteric methane emissions, which are a major source of greenhouse gases in livestock production, could be good for the environment^37^.

The results indicate the potential of high-quality feed pellets to enhance ruminal fermentation profiles in beef cattle. Enhancing the production of beneficial volatile fatty acids, such as propionate, while simultaneously reducing methane emissions through appropriate levels of pellet supplementation could improve the efficiency and sustainability of beef production. The balance between feed pellet concentration and its effects on various fermentation parameters requires careful management to achieve desired outcomes while maintaining animal health and performance.

**Table 4:** Effects of high-quality pelleted feed on BUN, HCT and rumen fermentation in beef cattle.

Items	Level of pellet feed in diet (%)				SEM	Contrast	
	0	20	40	60		Linear	Quadratic
BUN, mg/dl	12.55	12.96	12.51	12.68	0.37	0.77	1.00
HCT, %	28.11	29.00	28.78	29.67	1.09	0.31	0.91
Ruminal, pH	6.80	6.75	6.65	6.69	0.07	0.23	0.43
Ruminal temperature, ^o^C	38.78	39.33	38.63	39.06	0.62	0.97	0.90
NH_3_-N, mg/dl	9.15^c^	11.64^b^	12.53^b^	14.21^a^	0.49	< 0.01	0.42
Total VFA, mmol/l	107.67^b^	108.91^b^	109.13^b^	111.41^a^	0.69	0.01	0.48
	VFA profiles, mol/100mol						
Acetic acid	64.47^a^	59.51^b^	59.54^b^	56.52^b^	1.16	0.01	0.42
Propionic acid	25.80^b^	29.82^ab^	29.07^ab^	32.64^a^	1.46	0.01	0.88
Butyric acid	9.73^b^	10.68^ab^	11.39^a^	10.85^ab^	0.45	0.08	0.13
CH_4_ production^A^, mol/100mol TVFA	25.81^a^	22.85^ab^	23.35^ab^	20.79^b^	1.04	0.01	0.85

^ab^ Values in the same row with different superscripts differ (*P* < 0.05).

SEM = standard error of the means; ^A^Calculated according to Moss et al. (2000) CH_4_ production = 0.45 (acetate) − 0.275 (propionate) + 0.4 (butyrate).

Table 5 illustrates the impact of concentrate-related dietary supplementation on the microbial population in the rumen of beef cattle. Research indicates that dietary supplements in conjunction with concentrates do not influence fungal or bacterial populations. Supplemental diets with concentrates resulted in statistically significant decreases in protozoa populations as pellet levels increased. A previous study reported that cassava pulp and cassava chips contain condensed tannins, which may affect protozoa populations. Cherdthong^38^ indicates that condensed tannins from plant substances can inhibit protozoan growth and decrease the population of methane-producing microorganisms. A mechanism for the removal of protozoa may exist. Tannins have the ability to penetrate protozoan cell membranes, leading to their destruction, which is a critical factor in the elimination of protozoa. When dairy cows included samanea saman meal in their diet, they observed a reduction in protozoa numbers, similar to the findings of Anantasook et al.^39^, who investigated the impact of plants with secondary compounds resistant to palm oil on the edibility, digestibility, microbial protein synthesis, and microbial population.

However, the total bacteria, *F. succinogenes*, *R. flavefaciens*, and *R. albus* did not significantly change among the treatments according to a microbial population analysis conducted using real-time PCR (*P* > 0.05). Conversely, there was a decrease in protozoa in beef cattle fed powdered Manila palm (*P* < 0.05). However, Russell and Rychlik^40^ reported that microbial ecology varies depending on the feed given to ruminants. Previous studies by Poungchompu et al.^41^ demonstrated that the addition of MPP to soapberry fruit strongly reduced the amount of rumen protozoa. Herbs high in tannins can also lower protozoa^42^. The majority of *F. succinogenes* populations were greater in the rumen digesta than in the rumen fluid, according to Wanapat and Cherdthong^43^. On the other hand, microbial populations were unaffected by 3% mangosteen peel supplementation. Salais et al.^44^ stated that a person’s response to a diet can be strongly influenced by the kind of fiber and protein content of the forage. It is widely acknowledged that NH_3_ is required for cellulolytic bacterial development. The plants could not grow on other nitrogen sources without NH_3_. The fundamental explanation for the observed increase in attached cell counts could be attributed to cell growth on the straw, according to the authors of the study by Koike et al.^45^. However, the number of connected cells of all three species increased gradually, peaking at 24–48 h (10^9^ attached cells per gram of dry matter (DM) for *F. succinogenes*, 10^7^ attached cells per gram of DM for *R. flavefaciens*, and 10^6^ attached cells per gram of DM for *R. albus*). Four hours after feeding, there may have been additional bacterial adhesion from the liquid phase or other particles, in addition to postmeal cell development.

The effects of dietary supplementation, particularly when combined with concentrates, on the microbial population in the rumen of beef cattle have been a subject of interest due to their implications on animal health and productivity. According to the data presented in Table 5, research has demonstrated that while dietary supplements paired with concentrates do not significantly affect the fungal or bacterial populations in the rumen, they do lead to a reduction in protozoa populations, especially as pellet levels increase. This suggests that the concentrate form of supplementation may selectively influence protozoal abundance without altering the populations of total bacteria, *Fibrobacter succinogenes*, *Ruminococcus flavefaciens*, or *Ruminococcus albus*, as confirmed by microbial population analysis using real-time PCR (*P* > 0.05). There was a statistically significant drop in protozoa populations (*P* < 0.05) when beef cattle were fed powdered Manila palm. This shows that certain supplements can selectively affect certain microbial groups in the rumen. The decrease in protozoa may enhance nutrient utilization efficiency by diminishing protozoal predation on bacteria, which could subsequently improve overall rumen fermentation and animal performance. Jiao et al.^46^ conducted a study on the impact of several dietary supplements on the rumen microbiota, which provides more in-depth knowledge. The results of this study were similar. They showed that protozoa were more affected by changes in food supplements, especially when there were higher concentrate levels and certain additives like Manila palm. Bacterial populations stayed mostly the same.

**Table 5:** Effects of high-quality pelleted feed on the rumen microbial population of beef cattle.

Items	Level of pellet feed in diet (%)				SEM	Contrast	
	0	20	40	60		Linear	Quadratic
Total direct count (cells/mL)							
Protozoa (×10^5^)	4.89^a^	4.77^ab^	4.58^b^	4.55^b^	0.09	0.01	0.55
Anaerobic fungi (×10^7^)	6.68	6.66	6.64	6.66	0.03	0.44	0.46
Bacteria (×10^11^)	5.42	5.42	5.42	5.48	0.05	0.47	0.52
Real-time PCR technique, copies/ml of rumen content							
Total bacteria, ×10^9^	15.18	24.22	18.18	20.21	3.01	0.41	0.27
Protozoa, ×10^6^	7.59^a^	5.99^ab^	5.71^ab^	4.39^ab^	0.66	0.02	0.85
*F.succinogenes*, ×10^6^	5.66	5.33	5.39	5.26	0.87	0.85	0.81
*R.flavefaciens*, ×10^6^	26.61	28.61	30.85	28.43	3.29	0.61	0.52
*R.albus*, ×10^6^	4.15	3.37	4.15	3.30	1.34	0.83	0.74

^ab^ Values in the same row with different superscripts differ (*P* < 0.05).

*SEM*  Standard error of the means.

Table 6 illustrates the impact of feeding supplements in conjunction with concentrated feed on microbial protein synthesis in beef cattle. The current study showed no significant difference in the release of purine derivatives, but it did enhance the absorption of nitrogen derivatives by microorganisms and the efficiency of microbial protein synthesis in high-quality beef cattle pellets compared to other high-quality beef cattle pellets. The results are consistent with studies by Khampa et al.^47^ and Gunun et al.^48^, which showed that fermenting rice straw with urea enhanced its edibility, nutrient digestibility, rumen fermentation, and the efficiency of microbial protein synthesis. The impact of feeding supplements alongside concentrated feed on microbial protein synthesis in beef cattle is essential for enhancing livestock productivity. The findings in Table 6 reveal that the release of purine derivatives, a key marker for microbial protein synthesis, did not exhibit a significant difference. However, incorporating high-quality beef cattle pellets into the diet significantly increased both the absorption of nitrogen derivatives by microorganisms and the efficiency of microbial protein synthesis.

This indicates that, although the release of purine derivatives is stable, the application of high-quality pellets improves nitrogen utilization by rumen microbes. The enhancement of nitrogen absorption efficiency facilitates improved microbial growth and increased protein synthesis, both of which are essential for the overall growth and productivity of cattle. The enhancement in microbial protein synthesis efficiency indicates that cattle can convert feed into muscle mass more effectively, thereby improving the production of high-quality beef. A study by Firkins et al.^49^, which investigated the connection between microbial protein synthesis and meal quality, came to a similar conclusion. The study discovered that better diets, especially those high in concentrated feed and supplements, encouraged rumen bacteria to better utilize nitrogen, which in turn boosted microbial protein synthesis. This subsequently encouraged better growth performance in beef cattle.

**Table 6:** Effects of high-quality pellet feed on microbial protein synthesis in beef cattle.

Items	Level of pellet feed in diet (%)				SEM		Contrast		
	0	20	40	60			Linear		Quadratic
Microbial protein synthesis									
PD excreted, mmol/d	24.79	26.07	26.52	26.74	0.76		0.10		0.50
PD absorbed, mmol/d	76.39^b^	87.27^a^	90.62^a^	92.54^a^	2.83		0.01		0.15
MNS, gN/d	55.54^b^	63.45^ab^	65.88^a^	67.28^a^	3.06		0.02		0.32
EMPS, gN/kg OMDR	21.77^b^	28.71^ab^	27.70^ab^	30.80^a^	2.28		0.03		0.42

^ab^ Values in the same row with different superscripts differ (*P* < 0.05).

*SEM * Standard error of the means, *PD* purine derivative, *MNS * microbial nitrogen supply, *EMPS*  efficiency of microbial protein synthesis, *OMDR* organic matter digestible in the rumen.

## Conclusion

The present study concluded that pellet feeding in conjunction with concentrated feed at 60% resulted in decreased methane production, protozoa population and nutrient digestibility, microbial protein synthesis, total volatile fatty acids and propionic acid.

## Data Availability

Yes, All relevant raw data will be freely available from the authors and should be contacted “Thitima Norrapoke” if someone wants to request the data from this study.
